# Experimental Investigation of Generating Laser Beams of on-Demand Lateral Field Distribution from Digital Lasers

**DOI:** 10.3390/ma12142226

**Published:** 2019-07-10

**Authors:** Cing-Yi Huang, Kuo-Chih Chang, Shu-Chun Chu

**Affiliations:** Department of physics, National Cheng kung University, No.1, University Road, Tainan City 701, Taiwan

**Keywords:** digital lasers, structure beams, beam shaping, spatial light modulator, multi-point laser beam, arbitrary beam

## Abstract

A new type of laser system, known as a digital laser, was proposed in 2013. Many well-known laser beams with known analytical forms have been successfully generated in digital lasers. However, for a light field that does not have an analytical form, such as a multi-point light field or a light field with an arbitrary lateral distribution, how to generate such a light field from a digital laser has not been explored. The goal of this study was to experimentally explore how to generate an on-demand lateral laser field in a digital laser. In this study, a multi-point Gaussian laser beam was successfully generated in a digital laser by both controlling the range of the laser gain and the modulation of the phase boundary of the end of the cavity. This study then generated laser beams with an on-demand lateral field distribution by generating a superimposed multi-point laser field in a digital laser. Examples of triangles, rectangles, and letter T-shaped light fields produced by digital lasers were experimentally demonstrated. In summary, this study experimentally showed that a laser beam with an on-demand lateral field distribution could be generated in a digital laser by generating a superimposed multi-point laser field in a digital laser, in which a laser gain region covering the entire intra-cavity multi-point light field and the projected SLM (spatial light modulator) modulation function adopting a mimic amplitude mask are both used.

## 1. Introduction

Structural beams, which are light beams with a specific transverse field distribution, have been widely used in areas such as lithography [1,2,3], photopolymerization [4,5,6,7,8,9,10], optical tweezers [11,12,13], and other applications. The beam shaping method is commonly used to generate structured beams. The beam shaping method can be divided into two types, i.e., intra-cavity and extra-cavity beam shaping. Extra-cavity beam shaping achieves beam shaping by passing light beams through passive components such as refractive/diffractive optical elements [14,15,16,17] or spatial light modulators with optical phase or optical amplitude modulations [18,19]. Intra-cavity laser beam shaping is mainly accomplished by placing refractive/diffractive optical elements in the laser cavity, so as to modulate either the phase and/or the amplitude of the oscillating light field in the laser cavity [20,21,22,23,24,25,26]. Overall, most of the optical elements used for intra-cavity beam shaping must be specially designed for the specified lasering light field. This is not as good as an SLM (spatial light modulator) or DMD (digital mirror device), which are commonly used in external cavity beam shaping to dynamically control the lasering light field. Therefore, scholars have tried to incorporate adjustable optical components such as bimorph mirrors [20], LC (liquid-crystal) light valves [21], and DMD deformable mirrors [22,24] into the resonant cavity for immediate intracavity laser shaping/selection. However, the aforementioned methods still have significant limitations on the selection capability of the intracavity laser mode due to the pixel or spatial resolution limitation of the introduced optical modulation element. 

Recently, due to the improvement of technology, the increase in pixel number, diffraction efficiency, and damage threshold of the SLM have made it possible to incorporate SLM components into the laser cavity to achieve instant intracavity beam shaping [25,26,27,28]. Sandile Ngcobo and other scholars have proposed a new laser system, known as a digital laser, that uses the SLM as part of the laser cavity. By adjusting the phase modulation of the SLM, the phase or amplitude boundary of the digital laser can be manipulated in real time. Sandile Ngcobo and other scholars have successfully generated a number of well-known laser beams with known mathematical forms in digital lasers, such as Hermite-Gaussian, Laguerre-Gaussian, Airy beams, and other modalities [25]. Indeed, the production of laser beams with a known mathematical form in a digital laser requires only that the loading of the SLM corresponds to the required phase boundary of the specified laser beams. However, researchers are curious about how to generate laser output using an arbitrary transverse light field that does not have an analytically-resolved mathematical form. Through simulation of a laser converging light field, the authors previously predicted that it would be possible to produce multi-point laser beams from digital lasers and generate laser beams with an on-demand lateral field distribution through superimposing intra-cavity multiple point laser beams [29]. 

The aim of this study was to confirm the feasibility of the proposed method [29] through experiments as well as find the specific experimental operation method of generating laser beams with an on-demand lateral field distribution from digital lasers. In summary, this study experimentally shows that a laser beam of on-demand lateral field distribution can be generated in digital laser by generating a superimposed multi-point laser field in digital laser, in which a laser gain region covering the entire intra-cavity multi-point light field and the projected SLM modulation function adopting an amplitude mask are both used in the digital laser system. The results of this research will be beneficial to extensive areas, such as material processing, lithography, photopolymerization, and optical tweezers among other applications.

## 2. Experimental Setup and Method 

### 2.1. Experiment Setup 

**Figure 1:**
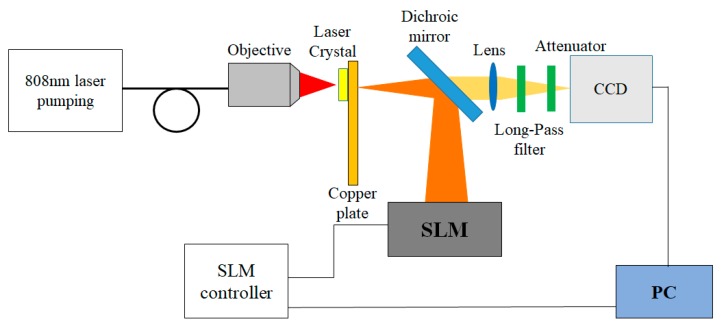
Experiment setup. The incident surface of the laser crystal, the 45-degree-tilted dichroic mirror, and the reflective panel of the SLM formed a folded L-shaped laser cavity. The dichroic mirror was the output coupling mirror of the laser system. The output laser beam passed through the lens, a long pass filter, and an attenuator, and was then observed on the CCD.

First, the digital laser system architecture used in this study is described. Figure 1 shows the digital laser setup used in this experiment, which consisted of an end-pumped solid-state laser. The 808 nm pumping light source was focused by a microscope objective lens (20x, numerical aperture 0.4) onto a 5 × 5 mm^2^, 1 mm thick, 3%-doped, a-cut Nd:GdVO_4_ crystal attached with heat-dissipating adhesive to a copper sheet that had a 3 mm hole. The microscope objective was mounted on a three-axis stage to control the range and the position of the intra-cavity laser gain. The direction of the a-cut Nd:GdVO_4_ laser crystal allowed the polarization direction of the resulting laser light to exactly match the polarization direction of the SLM demand. The incident surface of the laser crystal (transmission at 808 nm > 95%; reflectance at 1064 nm > 99.8%), the 45-degree-tilted dichroic mirror, and the reflective panel of the SLM formed a folded L-shaped laser cavity. A dichroic mirror (transmission at 808 nm > 85%; reflectance at 1064 nm > 97%) was used as the output coupling mirror of the laser system. The output laser beam passed through the lens, a long pass filter, and an attenuator, and was then observed on a CCD (charge-coupled Device). The spatial light modulator used in the experiment was a phase modulator, which was a commercial product (LCOS-SLM X13138-03WR) produced by Hamamatsu Photonics of Japan. The main specifications were as follows. The size of the liquid crystal panel was 16 × 12.8 mm, the single pixel size was 12.5 × 12.5 μm^2^, the pixel number was 1280 × 1024, the phase from 0 to 2π was divided into 216 steps, the fill factor was 96%, and the light adopting efficiency was 97%. In the experiment, the pumping light source adopted the mechanism of a quasi-continuous pump (frequency = 100 Hz, pulse width = 285 μs). The purpose of using quasi continuous pumping was to reduce the thermal effects of the crystal and to avoid possible damage to the laser crystal under the tight focus of the pumping light source. 

### 2.2. Method 

The experimental procedure is described below. In the experiment setup, the laser crystal, dichroic mirror, and SLM reflective panel formed a plano-concave-like cavity. The specified multi-point laser field was composed of a plurality of lowest order mode Gaussian beam arrangements. The optical waist position of the multi-point laser field in the cavity was located on the incident surface of the laser crystal, i.e., z = 0. First, the multi-point laser field U(z = 0) at the waist was specified. Next, the light field propagated to the SLM, U(z = L) = A(z = L)exp(i*ϕ*(z = L)), was numerically calculated by Fresnel–Kirchhoff integration [30]:

(1) $$\begin{array}{l} U\left(x_{0},y_{0},z=L\right)=\frac{\exp\left(jkL\right)}{j\lambda L}\\ \times \iint_{}U\left(x_{i},y_{i},z=0\right)\times \exp\left\{\frac{jk}{2L}\left[{\left(x_{i}-x_{0}\right)}^{2}+{\left(y_{i}-y_{0}\right)}^{2}\right]\right\}dx_{i}dy_{i} \end{array}$$

where, L represents the effective length of the laser cavity (i.e., the optical path length from the incident plane of the laser crystal to the SLM panel), *λ* is light wavelength, *j* is the unit imaginary number, *k* is the wave number of light field, and (*x*_i_, *y*_i_) and (*x*_0_, *y*_0_) are transverse coordinates of the z = 0 and z = L plane, respectively. Next, the phase projected onto the SLM was calculated based on the calculated U(z = L). And finally, the lateral light field distribution of the laser output was then observed on the CCD. 

The design of the SLM modulation function of this study was explained as follows. The SLM modulation function of this study was M(z = L) = P exp(i*ϕ*_SLM_), where *ϕ*_SLM_ = −2 *ϕ*(z = L) and P is the amplitude modulation function of the SLM. The projected phase on the SLM, *ϕ*_SLM_ = −2 *ϕ*(z = L), caused the reflected light field of the specified multi-point laser field to become the conjugate light of the incident light field; that is, the reflected light field and the incident light field differed only in the propagation direction. Through using the SLM to adjust the phase boundary of the resonant cavity, the resonant cavity could become a stable resonant cavity of the specified multi-point laser light field. The effect of function P on modulation function M was similar to an amplitude mask, and its function value was 0 or 1. This included the effect of a lateral truncation of the reflective light wave at the SLM panel. The purpose of P was to diffract out the portion of the intra-cavity light field that did not belong to the specified laser field. The spatial distribution value of the P function was given by the calculated multi-point light field propagating to the SLM, U(z = L). The portion having a much lower intensity than the peak intensity of the calculated specified field U(z = L) was set to 0 and the rest was set to 1. The experimental operation of adding an amplitude mask function P to function M was to add a phase distribution with a checkerboard pattern [25] to the portion of the P-function value of 0 in the phase modulation function. In this way, the probability that the light field of a non-specified structure would survive in the laser cavity could be reduced, so as to strengthen the light field selection ability of the laser cavity. In this study, the effective length L of the laser cavity used was 11.30 cm and the cutoff boundary of the P function was 4% of the peak intensity of the light field U(z = L). By using the phase boundary M design described above, the non-specified structure light field in the intracavity light field would gradually diffract out of the cavity after repeated round-trip propagation, and the light field in the cavity would gradually converge to the specified multi-point laser light field. 

This study first verified the feasibility of generating multi-point laser output in a digital laser by an experiment [29], and then generated a superimposed multi-point laser distribution in a digital laser to produce the desired specified lateral distribution of the laser output. In this study, a multi-point laser was generated in a digital laser by making the laser cavity become a stable cavity for only the specified multi-point laser beam through the control of the phase boundary of the laser cavity by SLM. Thus, the cavity enabled the spontaneous radiation to converge into a specified multi-point laser field through multiple oscillations in the laser cavity. Next, this study approximated an on-demand transverse light field by superposition of a multi-point laser. A multi-point laser with a specified distribution was then generated in a digital laser using the multi-point laser generation method described above. That is, a laser output with an on-demand transverse field strength distribution was generated in a digital laser.

## 3. Results and Discussion

Section 3.1 presents the results of generating a multi-point laser beam in a digital laser, and Section 3.2 presents the results of generating a laser beam with an on-demand transverse field in a digital laser. The effect of using the amplitude mask projected on the SLM, i.e., the P function, to the experiments is addressed in Section 3.3.

### 3.1. Generating a Multi-Point Laser Beam 

#### 3.1.1. The Influence of Spacing between Two-Point Beams

**Figure 2:**
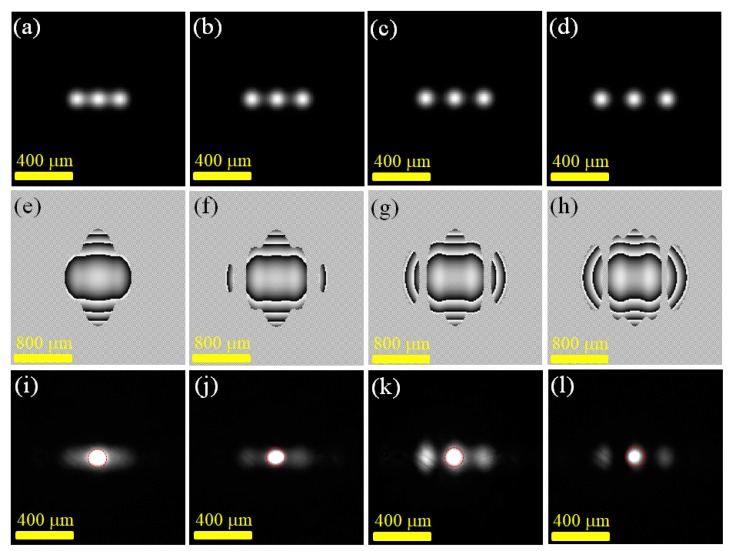
Experiments of generating 3-point light fields with different two-point spacings (*d*): (**a**)–(**d**) Calculated intensity, *d* = 150, 175, 200, and 225 μm, respectively; (**e**)–(**h**) SLM projected phase diagram when *d* = 150, 175, 200, and 225 μm, respectively; (**i**)–(**l**) Resulting field distribution when *d* = 150, 175, 200, and 225 μm, respectively.

Figure 2 shows the experimental results of generating three-point beams with different two-point spacings. Figure 2a–d plot the three-point light field distribution specified at z = 0, i.e., U(z = 0). The beam waist, w_0_, of each single-point Gaussian beam was 75 μm. The two-points spacings, *d*, shown in Figure 2a–d were 150, 175, 200, and 225 μm, respectively. Figure 2e–h are the phase distributions projected on the SLM projection for generating the specified three-point laser distribution in Figure 2a–d. Figure 2i–l are the laser output light field intensity distributions corresponding to the specified laser distribution shown in Figure 2a–d, respectively. In this experiment, the pumping beam was focused at the central spot position, in which the dotted red circle in Figure 2i–l indicates the gain range of the laser. The experimental results showed that when the preset two-point spacing of the light field gradually decreased, the experimentally-obtained light field gradually disappeared in the dark between each of the two points. Furthermore, it could be clearly seen from Figure 2i–l that the field intensity at the laser gain range of the output laser field was significantly higher than that at the other ranges. If it was desired that the laser field strength output at each point not be affected by the gain range, it was speculated that the entire field strength distribution range would be covered in the gain range.

#### 3.1.2. The Influence of the Laser Gain Range Size 

**Figure 3:**
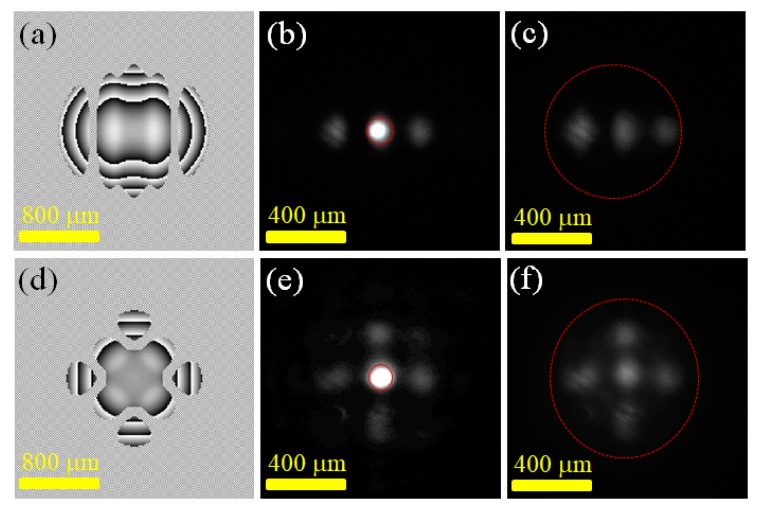
Experiments of generating three-point and five-point light fields with different gain regions: (**a**) SLM projected phase diagram for generating a 3-point laser; (**b**) the resulting field distributions when a small gain range is adopted; (**c**) the resulting field distributions when a large gain range is adopted; (**d**) the SLM projected phase diagram for generating a 5-point laser; (**e**) the resulting field distributions when a small gain range is adopted; (**f**) the resulting field distributions when a large gain range is adopted.

Based on the experimental results shown in Section 3.1.1, this study further explored the relationship between the laser gain region and the field intensity distribution of a multi-point laser output. In the experiment, the effective laser gain region of the laser crystal could be adjusted by changing the longitudinal position of the microscope objective along the light propagation direction. Figure 3 presents the intensity distribution of a multi-point laser output when the digital laser changed the laser gain region while using the same phase boundary. Figure 3a–f are the results for generating a three-point Gaussian beam and a five-point Gaussian beam, respectively. Figure 3a,d are the projected phase distribution diagrams on the SLM in the experiment. The four graphs of Figure 3b–e are the field intensity distributions of the three-point and five-point laser outputs obtained by the experiments, in which the red dotted circle indicates the distribution of the laser gain range in each experiment. In this experiment, the single-point Gaussian beam waist w_0_ was 75 μm and the spacing between the two points *d* was 225 μm. The maximum to minimum peak intensity ratio of the three/five peaks shown in Figure 3b,c,e,f were 3.01, 1.51, 9.11, and 1.93, respectively. The experimental results showed that the output point laser could obtain a relatively uniform field strength distribution when the entire multi-point beam field was covered in the gain range as compared to the use of the tight focused pumping gain region. It is worth noting that when using a larger area of laser gain range, the laser threshold of the digital laser would increase. At this time, it would be necessary to increase the pumping beam power to achieve multi-point beam lasering. Taking this experiment as an example, the power of the pumping beam in the four experiments (Figure 3b,c,e,f) were 0.37 mW, 0.65 mW, 0.37 mW and 0.65 mW, respectively. Using a wide region of laser gains (Figure 3c,f) required a higher pumping beam power than when using a small range of laser gains (Figure 3b,e). The experiments showed that with the control of both laser gain range and the end-side phase boundary, a multi-point Gaussian laser beam with an on-demand spatial distribution could be generated from a digital laser. The research results could be applied to the fabrication of three-dimensional microstructures, such as local electrophoresis depositions [4].

### 3.2. Generating an on-Demand Lateral Light Field with Multi-Point Beam Superposition 

This study approximated the on-demand transverse light field by superposition of a multi-point Gaussian laser beam and then generated a laser output with an on-demand transverse field strength distribution through generating a multi-point laser beam in a digital laser. 

**Figure 4:**
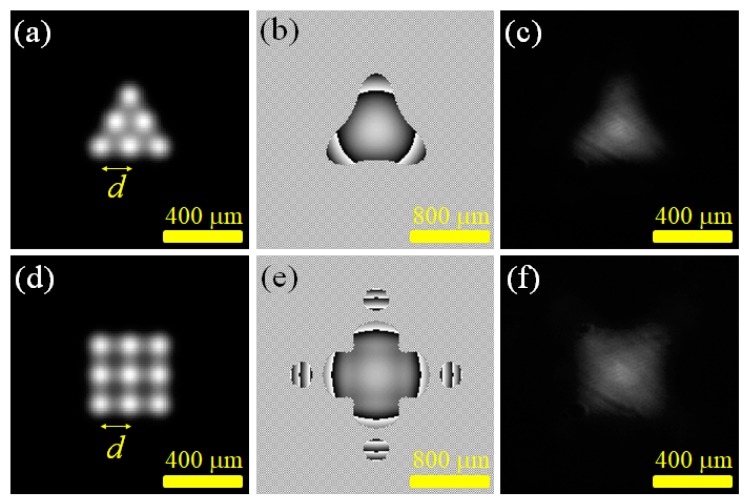
Experiments of generating triangular and rectangular laser light fields: (**a**) the calculated 6-point light field intensity; (**b**) the SLM projected phase diagram for generating a triangle light field; (**c**) the resulting triangle field distributions; (**d**) the calculated 9-point light field intensity; (**e**) the SLM projected phase diagram for generating a rectangle light field; (**f**) the resulting rectangle field distributions.

Figure 4 shows the experimental results of generating a laser field with a triangular and rectangular intensity distribution in a digital laser. In the experiment, the coherent superposition of 6-point and 9-point Gaussian laser beams was used to approximate the transverse field distribution of the triangular field and the rectangular field. Figure 4a,d plot the calculated U(z = 0) of the two experiments, which showed the lateral relative positions of the Gaussian beams of each point in the laser cavity at the z = 0 plane. In this experiment, the single-point Gaussian beam waist w_0_ was 75 μm and the spacing between the two points *d* was 150 μm. Figure 4b,e are the phase distributions projected on the SLM for generating the specified triangular field and the rectangular field, respectively. Figure 4c,f show the experimentally-obtained digital laser beam intensity distributions of the triangular field and rectangular field distributions. The experimental results confirmed that by using the method of generating a multi-point laser output in a digital laser, the laser output of the on-demand lateral field intensity distribution could be successfully generated according to user requirements. The threshold pump power of two lasers was measured to be 15.58 mW and 12.10 mW, respectively. From reaching the structure laser beam output to adjusting the pumping beam power to 150 mW, there was no significant change in the laser output light field pattern over the entire change in the pumping beam power. While taking the ranges surrounded by the peripheral points of the preset multi-point light field, shown in Figure 4a,c, as the comparison areas, the standard deviation values of the light intensity in the comparison areas shown in Figure 4a,c,d,f were 0.18, 0.11, 0.19, and 0.05, respectively. The two output transverse fields obtained in the experiment, shown in Figure 4c,f, had a higher uniformity of intensity distribution over the preset triangular/rectangular range compared to the preset light field, shown in Figure 4a,d. The on-demand lateral light field generated in this study could be applied to the research area of material processing. For example, a rectangular light field could be used to fabricate three-dimensional structures [5] and microscale medical devices [6].

**Figure 5:**
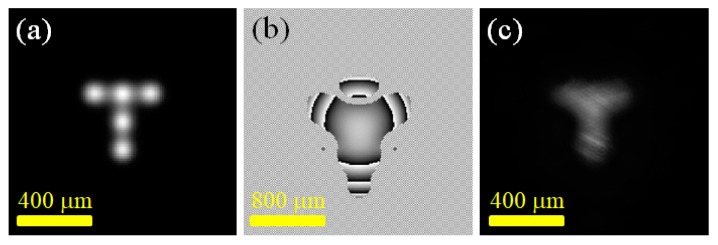
Experiments of generating a T-shaped laser light field: (**a**) the calculated 5-point light intensity; (**b**) the SLM projected phase diagram; (**c**) the resulting laser light field distributions.

Figure 5 presents the experimental results of using a digital laser to produce a transverse field strength distribution in the shape of the letter “T”. In the experiment, the coherent superposition of five Gaussian laser beams was used to approximate the transverse field distribution of the shape. Figure 5a plots the calculated U(z = 0) of the experiment, which showed the lateral relative positions of the Gaussian beams of each point in the laser cavity at the z = 0 plane. In this experiment, the single-point Gaussian beam waist w_0_ was 75 μm and the spacing between the two points *d* was 150 μm. Figure 5b is the phase distribution projected on the SLM for generating the specified T-shaped field. Figure 5c shows the experimentally-obtained digital laser beam intensity distributions. The experimental results again confirmed that by using the method of generating a multi-point laser output in a digital laser, a laser output with an on-demand lateral field intensity distribution could be successfully generated according to user requirements. The success in generating a lateral light field in the shape of the letter “T” showed the potential of this study for the laser writing of micro-optical and photonic structures [7]. 

### 3.3. Discussion: The Influence of Using Amplitude Mask

**Figure 6:**
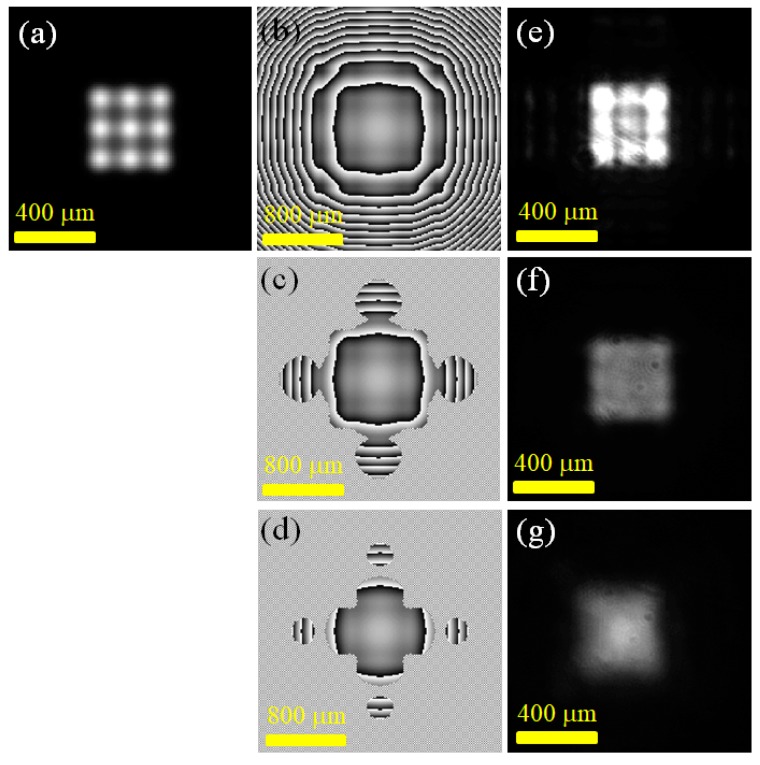
Experiments of generating a rectangular light field using amplitude masks with different truncated ranges: (**a**) the calculated 9-point light intensity; (**b**) the SLM projected phase diagram in which no amplitude mask is used; (**c**) the SLM projected phase diagram in which the mask cuts off a smaller range of light fields; (**d**) the SLM projected phase diagram in which the mask cuts off a larger range of light fields; (**e**) the resulting light field distribution in which no amplitude mask is used; (**f**) the resulting light field distribution in which the mask cuts off a smaller range of light fields; (**g**) the resulting light field distribution in which the mask cuts off a larger range of light fields.

From the results of the experiment in Section 3.2, it could be found that the output multi-point laser field obtained by the experiment was obviously different from the calculated coherent superimposed field of the multi-point Gaussian beam. Compared with the calculated multi-point coherent superposition light field distribution, the experimentally-obtained laser output had a significantly more uniform field intensity distribution within the specified range. This result could be explained by the diffraction effect caused by the amplitude mask introduced in the SLM modulation function. This study added an amplitude mask, i.e., the P function, in the modulation of the SLM (M). Experimentally, it was found that when the amplitude mask was added, the size of a single spot of the generated multi-point laser would increase. Figure 6 shows the effect of changing the amplitude mask range of the phase modulation function of the SLM in the experiment of producing a rectangular light field. Figure 6a is the calculated 9-point Gaussian beam intensity distribution at z = 0. Figure 6b–d plot the projected phases on the SLM using different ranges of amplitude masks. In Figure 6b–d, the boundary of the P function value (0, 1) was set as 0%, 0.25%, and 4% of the peak value of the calculated propagating the field intensity at the SLM, |U(z = L)|^2^, respectively. The standard deviation values of the light intensity in the rectangular comparison areas shown in Figure 6e,f,g were 0.10, 0.07, and 0.02, respectively. The experimental results showed that with the increase of the truncated range of the amplitude mask, the distribution of the experimental laser output field became more uniform in the specified rectangular range. This phenomenon could be explained by the fact that the wavefront range was cut off by the amplitude mask (P limits the effective aperture size of the laser system), causing the size of the single Gaussian spot to expand due to the diffraction effect. When the single-point expansion effect was applied to the generation of an on-demand light field with a multi-point laser superposition approximation, a more uniform light field distribution could be obtained.

## 4. Conclusions

This study experimentally demonstrated that a laser beam with an on-demand lateral field distribution could be generated by a digital laser and provided a method for implementation. That is, a laser beam with an on-demand lateral field distribution could be generated in a digital laser by generating a superimposed multi-point laser field in digital laser, in which a laser gain region covering the entire intra-cavity multi-point light field and the projected SLM modulation function adopting a mimic amplitude mask are both used. The results of this research could be beneficial to numerous areas, such as material processing, lithography, photopolymerization, and optical tweezers among other applications.
